# Incidental Prostate Cancer in Patients Treated for Benign Prostatic Hyperplasia: Analysis from a Contemporary National Dataset

**DOI:** 10.3390/diagnostics14070677

**Published:** 2024-03-23

**Authors:** Eugenio Bologna, Leslie Claire Licari, Antonio Franco, Francesco Ditonno, Celeste Manfredi, Cosimo De Nunzio, Alessandro Antonelli, Marco De Sio, Costantino Leonardo, Giuseppe Simone, Edward E. Cherullo, Riccardo Autorino

**Affiliations:** 1 Department of Maternal-Child and Urological Sciences, Sapienza University Rome, Policlinico Umberto I Hospital, 00161 Rome, Italy; eugenio.bologna@uniroma1.it (E.B.); leslieclaire.licari@uniroma1.it (L.C.L.); 2 Department of Urology, Rush University, Chicago, IL 60612, USA; antonio.franco@uniroma1.it (A.F.); francesco.ditonno@icloud.com (F.D.); manfredi.celeste@gmail.com (C.M.); edward_cherullo@rush.edu (E.E.C.); 3 Department of Urology, Sant’Andrea Hospital, Sapienza University, 00189 Rome, Italy; cosimo.denunzio@uniroma1.it; 4 Department of Urology, Azienda Ospedaliera Universitaria Integrata Verona, University of Verona, 37129 Verona, Italy; alessandro_antonelli@me.com; 5 Unit of Urology, Department of Woman, Child and General and Specialized Surgery, University of Campania “Luigi Vanvitelli”, 80131 Naples, Italy; marco.desio@unicampania.it; 6 Department of Urology, “Regina Elena” National Cancer Institute, 00144 Rome, Italy; costantino.leonardo@gmail.com (C.L.); puldet@gmail.com (G.S.)

**Keywords:** BPH, incidental, MRI, prostate biopsy, prostate cancer

## Abstract

(1) Background: Prostate Cancer (PCa) may be incidentally diagnosed during the microscopic evaluation of resected tissue from BPH surgeries, characterizing the clinical condition known as incidental PCa (iPCa). This study aims to assess the prevalence of iPCa following BPH surgery to evaluate the associated surgical procedures and to scrutinize preoperative and postoperative management. (2) Methods: A retrospective analysis was conducted using the PearlDiver™ Mariner database, containing patient records compiled between 2011 and 2021. International Classification of Diseases (ICD) and Current Procedural Terminology (CPT) codes were employed to identify the population and outcomes. Our primary objective was to assess the prevalence of iPCa, categorized by the type of procedures, and to evaluate the subsequent treatment strategies. The secondary aim was to assess the impact of prostate biopsy (PB) and prostate MRI on iPCa detection. (3) Results: The overall cohort, accounting for 231,626 patients who underwent BPH surgery, exhibited a 2.2% prevalence rate of iPCa. The highest rate was observed for TURP (2.32%), while the lowest was recorded for RASP (1.18%). Preoperative MRI and PB demonstrated opposing trends over the years. Of the 5090 patients identified with iPCa, nearly 68% did not receive active treatment. The most common treatments were RT and ADT; 34.6% underwent RT, 31.75% received ADT, and 21.75% were treated with RT+ADT. RP was administered to approximately 9% of patients undergoing endoscopic procedures. Multivariate logistic regression analysis revealed age and openSP as additional risk factors for iPCa. Conversely, PB and MRI before surgery were linked to a decreased risk. (4) Conclusions: The contemporary prevalence of iPCa after BPH surgery is <3%. The increase in the use of prostate MRI mirrors a decline in the PB biopsy prior to BPH surgery but without resulting in an increased detection rate of iPCa. In contemporary routine clinical practice, iPCa is mostly managed in a different way when compared to biopsy-detected PCa.

## 1. Introduction

Prostate Cancer (PCa) may be incidentally detected and diagnosed during the microscopic evaluation of resected tissue from benign prostatic hyperplasia (BPH) surgeries, characterizing the clinical condition known as incidental PCa (iPCa). According to the most recent TNM classification [1], iPCa is classified as T1a tumor if the cancer is visible in less than 5% of the resected prostate, while T1b corresponds to cancer present in more than 5% of the resected tissue.

Over recent decades, enhanced PCa screening protocols, especially through prostate-specific antigen (PSA) testing [2], complemented by additional diagnostic tools [3] including multiparametric Magnetic Resonance Imaging (MRI) [4,5], have refined preoperative management strategies for patients awaiting BPH surgery. Moreover, new surgical techniques for BPH have advanced, with some techniques foregoing histopathological examination of prostatic tissue (e.g., photoselective vaporization of the prostate, robotic waterjet treatment, water vapor thermal therapy) [6]. These shifts in BPH management and treatment have contributed to the decreased detection rate of iPCa, considering that these treatments do not allow for a histopathological diagnosis of the prostate gland [7]. Nevertheless, a consistent number of patients—5–11% according to recent reports [8,9]—still receive a diagnosis of iPCa during histopathological examination after BPH surgery.

The diagnosis and management of iPCa require additional considerations compared to biopsy-detected PCa. Firstly, the transitional zone of the prostate—which represents the prostatic tissue resected during BPH surgeries—does not allow for an accurate estimation of the tumor’s extent and grading in comparison to a biopsy protocol that evaluates the peripheral zone, which is the most frequent site of PCa onset [10]. This raises additional challenges about the actual grading of the PCa and the potential need for an additional biopsy protocol to assess the true extent and grading of the tumor.

Furthermore, the diagnostic value of tumor-related specimens obtained via transurethral resection or enucleation is compromised by fragmentation and energy-induced damage. However, international guidelines do not delineate a specific management strategy for this unique diagnostic category, leading to unification under the pathological T1 (pT1) stage [10].

This study aims to assess the contemporary prevalence of iPCa following BPH surgery to evaluate the associated surgical procedures and to scrutinize both preoperative and postoperative management.

## 2. Materials and Methods

### 2.1. Dataset and Objectives

We conducted a retrospective analysis using the PearlDiver™ Mariner database (PearlDiver Technologies, Colorado Springs, CO, USA) [11]. It is a commercially available, all-payer national claims database, containing over 41 billion Health Insurance Portability and Accountability Act (HIPAA)-compliant patient records collected between 2011 and 2022. The dataset uses unique patient identifier codes, which allows for time-specific, longitudinal research, while also keeping patient information de-identified. 

Moreover, this resource catalogs healthcare interactions across inpatient and outpatient settings, facilitating the longitudinal study of patient trajectories. Coverage is comprehensive, extending to all payer models across the entirety of U.S. states and territories [12]. Specific International Classification of Diseases (ICD), both 9th (ICD-9) [13] and 10th (ICD-10) editions [14], and Current Procedural Terminology (CPT) codes were used to identify population and outcomes within the database. Data integrity is ensured via rigorous audits and review processes by independent third parties.

Our primary objective was to assess the prevalence of iPCa following BPH surgery and to evaluate the subsequent treatment strategies. The secondary aim was to assess the impact of prostate biopsy (PB) and prostate MRI on the detection of iPCa, as well as how their use has changed over time and affected iPCa detection rates. Additionally, we evaluated patient-related and procedure-related risk factors for the diagnosis of iPCa.

### 2.2. Study Population and Procedures

No institutional review board approval was needed for this study as the database contains de-identified data. We queried the database from 1 January 2011 to 31 December 2021 for all patients who underwent surgical procedures for BPH that entailed a histological evaluation after surgery.

BPH surgical approaches included in the study were as follows: Transurethral Resection of the Prostate (TURP), Holmium/Thulium Laser Enucleation of the Prostate (HoLEP/ThuLEP), Open Simple Prostatectomy (oSP), and Robot-Assisted Simple Prostatectomy (RASP).

Surgical procedures without a unique CPT code were excluded from this study. Moreover, our refined cohort included those with active insurance claims. We collected demographic variables including age and Charlson comorbidity index (CCI). We identified individuals who received a first diagnosis of PCa within 60 days following their BPH surgery, aiming to minimize the inclusion of PCa diagnoses that were not directly related to the BPH procedure. In this context, patients who had been previously diagnosed with PCa prior to BPH surgery were excluded to eliminate endoscopic procedures that were carried out with palliative intentions.

Moreover, we scrutinized the patient cohort that had received a PB and/or MRI within six months preceding the surgery. Our analysis extended to the post-diagnostic therapeutic management for iPCa, evaluating treatments, such as Radical Prostatectomy (RP), Radiation Therapy (RT), and Androgen Deprivation Therapy (ADT). 

Lastly, we explored potential risk factors linked to the diagnosis of iPCa.

### 2.3. Statistical Analyses

Upon identification of patients who underwent the BPH procedures of interest for our study, their baseline characteristics were extracted. Descriptive statistical variables were reported as frequencies and proportions for categorical variables, and as mean with standard deviation (SD) or median with interquartile range (IQR) for continuous variables. Prostate MRI and PB were reported as frequencies and proportions within the overall cohort and within each individual procedure; additionally, these frequencies and proportions were calculated for each respective year under study and depicted in a temporal-trends graph. The same approach was applied to the incidence rate of iPCa. 

The therapeutic trajectory following the diagnosis was—similarly—presented as frequencies and proportions, considering both single treatments and their various combinations. The sum of the individual interventions and their respective combinations allowed for the identification of patients who underwent active treatment following iPCa. Finally, multivariable logistic regression analysis was used to evaluate potential risk factors associated with iPCa.

## 3. Results

### 3.1. Operative and Preoperative Management

Following data extraction, 231,626 patients who underwent BPH surgery during the study period were identified. The baseline and preoperative characteristics are presented in Table 1.

**Figure 1:**
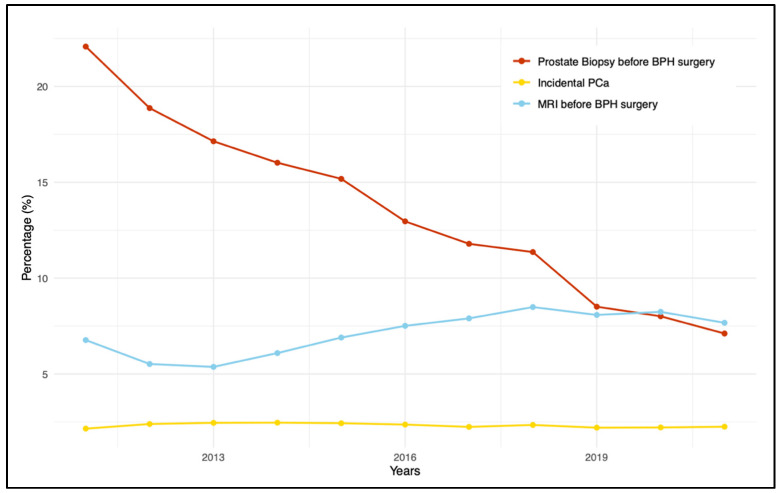
Trends in pre-surgical MRI, pre-surgical prostate biopsy, and detection of incidental PCA in BPH patients (2011–2021).

The most performed procedure was TURP, accounting for 197,146 cases (approximately 85% of the overall procedures). The least common was RASP, with 6362 procedures (2.75%). The percentage of patients undergoing PB in the six months preceding the surgery ranged from 6.16% for TURP to 13.06% for RASP. A similar trend was observed for prostate MRI, with the highest percentage associated with RASP (9.68%). Even considering the combination of the two methods (PB and MRI), RASP was associated with the highest utilization rates (4.32%). The trends of pre-surgical MRI and PB throughout the study period are depicted in Figure 1.

**Figure 2:**
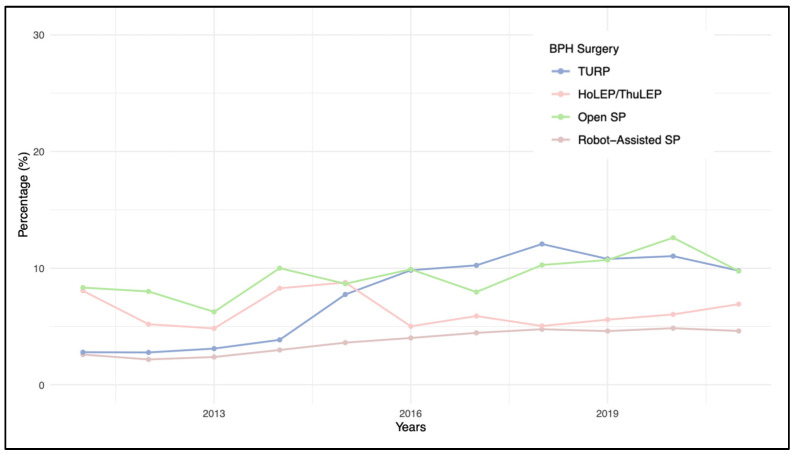
Pre-surgical MRI utilization trends for single procedure in BPH surgery patients (2011–2021).

**Figure 3:**
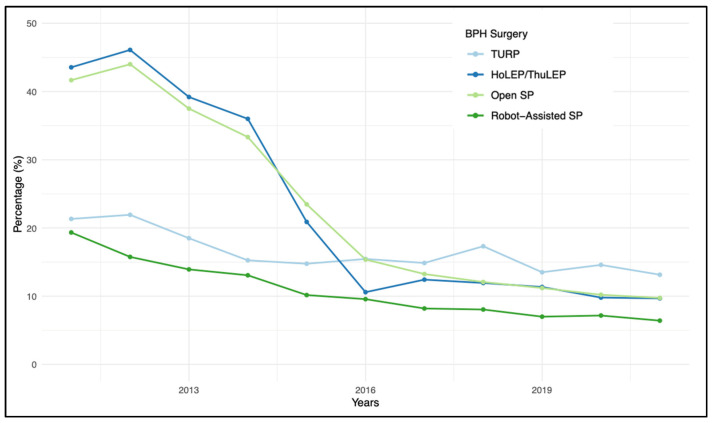
Pre-surgical prostate biopsy trends for single procedure in BPH surgery patients (2011–2021).

MRI and PB demonstrated opposing trends over the years; while prostate MRI showed a continuous increase—particularly from 2013 to 2018—PB exhibited a decreasing trend. The variations in trend utilization for MRI and prostate biopsy (PB), according to individual BPH procedures, are reported in Figure 2 and Figure 3, respectively.

### 3.2. iPCa and Subsequent Therapeutic Management

The overall cohort exhibited a 2.2% prevalence rate of iPCa (Table 2). The highest rate was observed in patients who underwent TURP (2.32%), while the lowest was recorded for those undergoing RASP (1.18%). The incidence rate of iPCa throughout the study period is illustrated in Figure 1. Among patients with iPCa diagnosis, the frequencies of MRI and PB utilization before surgery diminished, with observed rates ranging from 6.86 to 10.6% for PB and from 2.31 to 5.33% for MRI, respectively.

**Figure 4:**
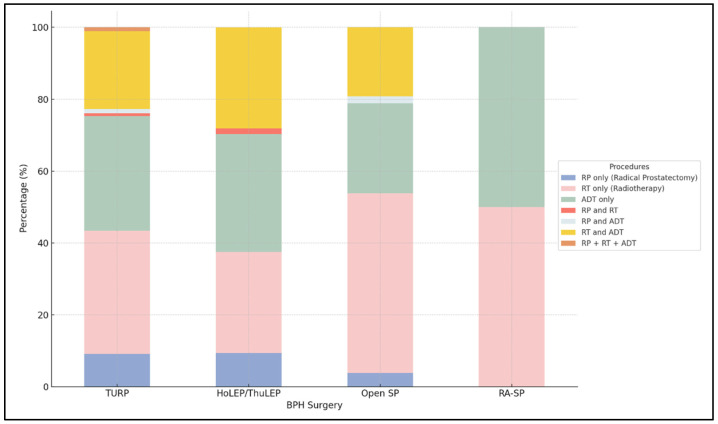
Treatment distribution for Incidental Prostate Cancer following initial BPH surgery procedures in actively treated patients.

Of the 5090 patients identified with iPCa, nearly 68% did not receive active treatment within the study period. Notably, patients undergoing RASP constituted the largest subgroup, with 94.67% not proceeding with post-diagnostic treatment. After iPCa, the most common treatments were Radiation Therapy (RT) and Androgen Deprivation Therapy (ADT). Specifically, 34.6% underwent RT, 31.75% received ADT, and 21.75% were treated with a combination of both modalities. RP, when used as only treatment, was administered to approximately 9% of patients undergoing endoscopic procedures, such as TURP and HoLEP/ThuLEP. In contrast, its utilization was lower following simple prostatectomy, including OSP and RASP (3.85–0%). The combined regimens of RP with RT, as well as the triad of RP, RT, and ADT, were the least employed treatment strategies. The percentage distribution of iPCa treatments post-BPH surgery is depicted in Figure 4.

### 3.3. Risk Factors for iPCa Following BPH Surgery

Multivariate logistic regression (MLR) analysis revealed age and open simple prostatectomy as additional risk factors for iPCa diagnosis (Table 3). Conversely, undergoing PB and MRI before surgery was linked to a decreased risk of iPCa. Specifically, pre-surgical prostate MRI was associated with an 18% risk reduction (OR (95% CI) = 0.82, (0.702–0.956), *p* = 0.011), and PB before surgery was correlated with a 26% risk reduction (OR (95% CI) = 0.74, (0.654–0.840), *p* < 0.001). The concurrent use of MRI and PB was linked to a significant decrease in iPCa risk by 39% (OR (95% CI) = 0.60 (0.42–0.85), *p* < 0.005). The HoLEP/ThuLEP procedures also correlated with a lowered risk of iPCa (*p* < 0.05).

A subgroup MLR analysis in patients with a preoperative screening with MRI or PB shows, conversely, no correlation for the individual procedures with iPCa detection (Table 4).

## 4. Discussion

The most notable finding from our study is the contemporary prevalence of iPCa following BPH surgeries. We observed a prevalence rate of 2.20%. This value is comparable to other studies with similar methodologies [15] yet is lower than other reports, which indicate rates between 5.6% and 23% [8,16,17,18]. It can be speculated that these variations in incidence rates could be attributed to several factors. 

First, it is reasonable to consider that single and multicentric studies assessing the incidence of iPCa have included postoperative histological examination as an inclusion criterion, which may be limited in national dataset studies. Another factor could be that most studies reporting higher rates originate from academic or tertiary referral centers; these institutions often employ specialized uropathologists, whose expertise could potentially influence the rate of diagnosis [19]. Finally, it could be asserted that academic or tertiary centers are associated with surgical volumes that may correlate with improved specimen quality after BPH surgery, thereby increasing the iPCa detection rate [20].

An intriguing finding is the utilization patterns of biopsy and MRI within the six months preceding the BPH surgical procedure. We recorded prevalence rates for MRI, PB, and their combination of 6.6%, 3.9%, and 1%, respectively. Despite the PB rate being nearly double that of MRI, the temporal analysis of these diagnostic methods throughout the study period showed diverging trends. On the one hand, there was a noticeable increase in the use of MRI. On the other hand, there was a significant decline in preoperative PB. Observed shifts in the use of these preoperative diagnostic tools did not alter the yearly incidence of iPCa. Arguably, the implementation of prostate MRI and of the PI-RADS classification system, which was introduced in 2012 with its first version [21] and subsequently updated in 2015 [22], allowed researchers to minimize the number of preoperative biopsies over time.

When considering individual procedures, RASP exhibits the highest preoperative utilization rates of MRI and PB. This could be attributed to the larger prostate volumes seen with this procedure, coupled with elevated PSA levels, leading to greater scrutiny for these patients. However, this does not account for the higher rates compared to HoLEP/ThuLEP and OSP. A potential explanation for this discrepancy could lie in the higher costs associated with robot-assisted procedures, which would not be justified if iPCa was found, possibly prompting a more thorough preoperative diagnostic approach [23].

In multivariable logistic regression analysis, PB and prostate MRI emerged as significant factors for reducing the risk of iPCa diagnosis, with the risk reduction reaching up to 40% when combining these two diagnostic modalities. Conversely, and in line with general epidemiological data, age was associated with an increased risk for iPCa [24]. 

Considering TURP as the reference, our regression analysis revealed OSP as a significant risk factor for iPCa (OR = 1.16, *p* = 0.02). The observed association between OSP and a higher risk of iPCa is possibly due to its frequent adoption in managing larger prostate volumes. Furthermore, the predominance of OSP in the early years of our study period, a time when MRI might have not become a widely used tool, could explain their greater numbers. Over time, the introduction of HoLEP/ThuLEP and RASP, which offer less invasive alternatives, likely contributed to the reduction in OSP [25]. In contrast, HoLEP/ThuLEP and RASP, which are also indicated for larger prostates, surprisingly showed a reduced iPCa risk. This protective effect might be influenced by the higher rates of preoperative MRI and PB in these patient groups, possibly leading to a selection bias that excludes higher-risk patients. This could explain why these procedures were associated with a lower risk of iPCa in our study compared to the existing literature. Indeed, in the subgroup analysis, including patients evaluated preoperatively with MRI and/or PB, the individual procedures no longer retained statistical significance as a risk factor for the detection of iPCa.

Moving on to the management of iPCa, further intriguing results emerged. Active treatment was pursued in 32.2% of iPCa patients. Within the subset of treated patients, RT and ADT were the most frequently administered treatments, representing 34.6% and 31.7% of cases, respectively. Conversely, RP, whether as a monotherapy or part of a combined treatment regimen, was used in 10% of patients receiving active treatment. These findings present intriguing perspectives: iPCa demonstrates a higher inclination towards RT compared to RP. This diverges from prevalent national patterns on biopsy-detected PCa, where RP is more commonly utilized, with rates ranging from 38% to 54.4% [26,27]. Almost 70% of patients did not undergo active treatment, confirming that iPCa is more commonly associated with conservative management [15].

There, thus, appears to be a general preference for the non-surgical approach in the management of iPCa. This may be partly explained by the patient’s or physician’s inclination to avoid additional surgical treatment in patients who have recently undergone surgery for BPH. However, there are also surgical considerations and related functional outcomes that must be considered. 

A recent meta-analysis conducted by Creta et al. reported that patients who underwent RP following BPH surgery exhibited higher rates of positive surgical margins, lower urinary continence rates at both 3-month and 1-year follow-ups, as well as diminished rates of erectile function recovery at 1-year follow-up [28]. In our study, the employment of RP is further reduced when considering patients who have undergone simple prostatectomy, whether open or robot assisted. This further reduction could be related to potential surgical challenges after prior abdominal interventions, although comparative studies do not indicate an increased risk of RP following abdominal surgery [29].

An additional finding from our study is the proportion of patients who have been subjected to ADT in monotherapy following iPCa diagnosis. A secondary analysis indicated that 182 patients (approximately 40% of this subgroup) developed bone metastases, thus justifying the therapeutic indication. However, there is a substantial proportion of patients for whom this therapy is not clinically justified. In 2010, a study conducted by Cooperberg et al. analyzed data from 11,892 men with biopsy-proven localized PCa from 36 clinical sites contributing to the Cancer of the Prostate Strategic Urologic Research Endeavor (CaPSURE) registry. The study results demonstrated that approximately 15% of the patients were treated with primary ADT [30], underscoring that a substantial proportion of the patient population is steered toward hormonal therapy without clear evidence of survival advantages [31]. It may be plausible to assume that this percentage could increase following a diagnosis of iPCa, using this treatment more as a palliative measure rather than therapeutic. If confirmed, this trend would shed light on a clinical practice associated with high management costs, in the absence of proven oncological benefits [32].

Our results on iPCa postoperative management highlight that, although guidelines do not indicate specific follow-up protocols or management strategies for T1a and T1b PCa—thus treating stage T1 as a clinically uniform entity—the therapeutic approach substantially deviates from that used for biopsy-detected PCa.

Our study is not without its limitations. The absence of detailed clinical and histological characterizations of prostate pathology precludes more accurate analyses of the real trends in both iPCa diagnosis and its management. Specifically, for iPCa, the lack of histological typing and more precise staging information impairs patient risk stratification. Regarding RT, despite the careful selection of associated CPT codes, the connection to prostate pathology is not robust, allowing for the possibility of overlap in the indication for RT due to other pelvic malignancies. Furthermore, it is not possible to ascertain the patterns and dosages of RT administered. Another inherent limitation of the PearlDiver Mariner database is the absence of data on patient race, tumor characteristics, and treatment protocols, which hinders a more thorough analysis of the examined outcomes. Nonetheless, we believe that the large sample size and the analyses performed on it define the actual management trends of iPCa.

## 5. Conclusions

Our findings indicate that the contemporary incidence rate of iPCa after BPH surgery is <3%. The increase in the use of prostate MRI mirrors a decline in the use of prostate biopsy prior to BPH surgery but without resulting in an increased detection rate of iPCa. In contemporary routine clinical practice, iPCa is mostly managed in a different way when compared to biopsy-detected PCa.

## Figures and Tables

**Table 1:** Baseline characteristics of patients undergoing BPH surgery and for individual BPH procedures during the study period.

	Overall BPH Surgery	TURP	HoLEP/ThuLEP	Open SP	Robot Assisted SP
**Procedures, n (%)**	231,626	197,146 (85.11)	18,169 (7.84)	9949 (4.30)	6362 (2.75)
**Age, mean (SD)**	71.72 (7.05)	71.57 (7.08)	71.48 (7.17)	72.38 (6.57)	71.63 (5.62)
**CCI, mean (SD)**	3.61 (2.94)	3.44 (2.85)	3.03 (2.22)	4.62 (3.52)	4.23 (3.05)
**PB before surgery (6 months), n (%)**	15,239 (6.58)	12,141 (6.16)	1473 (8.11)	794 (7.98)	831 (13.06)
**MRI before surgery (6 months), n (%)**	9035 (3.90)	6786 (3.44)	1150 (6.33)	483 (4.85)	616 (9.68)
**PB and MRI before surgery (6 months), n (%)**	2396 (1.03)	1616 (0.82)	362 (1.99)	143 (1.44)	275 (4.32)
**iPCa, n (%)**	5090 (2.20)	4575 (2.32)	233 (1.28)	207 (2.08)	75 (1.18)
**PB before iPCa (6 months), n (%)**	190 (3.73)	157 (3.43)	16 (6.86)	9 (4.34)	8 (10.6)
**MRI before iPCa (6 months), n (%)**	132 (2.59)	106 (2.31)	11 (4.72)	11 (5.31)	4 (5.33)
**PB and MRI before iPCa (6 months), n (%)**	21 (0.41)	17 (0.37)	2 (0.86)	1 (0.48)	1 (1.33)

Abbreviations: CCI (Charlson comorbidity index), PB (prostate biopsy), MRI (Magnetic Resonance Imaging), iPCa (Incidental Prostate Cancer).

**Table 2:** Distribution of active treatments (Radical Prostatectomy, Radiotherapy, and Androgen Deprivation Therapy) for Incidental Prostate Cancer after BPH surgeries.

	Overall BPH Surgery	TURP	HoLEP/ThuLEP	Open SP	Robot Assisted SP
**iPCa, n (%)**	5090 (2.20)	4575 (2.32)	233 (1.28)	207 (2.08)	75 (1.18)
**Patients with Active Treatment for iPCa, n (%)**	1641 (32.24)	1521 (33.25)	64 (27.47)	52 (25.12)	4 (5.33)
**Radical Prostatectomy (RP), n (%)**	147 (8.96)	139 (9.14)	6 (9.37)	2 (3.85)	0 (0)
**Radiotherapy (RT), n (%)**	568 (34.61)	522 (34.32)	18 (28.12)	26 (50.0)	2 (50.0)
**Androgen Deprivation Therapy (ADT), n (%)**	521 (31.75)	485 (31.89)	21 (32.81)	13 (25.0)	2 (50.0)
**RP + RT, n (%)**	12 (0.73)	11 (0.72)	1 (1.56)	0 (0)	0 (0)
**RP + ADT, n (%)**	19 (1.16)	18 (1.18)	0 (0)	1 (1.92)	0 (0)
**RT + ADT, n (%)**	357 (21.75)	329 (21.63)	18 (28.12)	10 (19.23)	0 (0)
**RP + RT + ADT, n (%)**	17 (1.04)	17 (1.12)	0 (0)	0 (0)	0 (0)
**Patients w/o Active treatment, n (%)**	3449 (67.76)	3054 (66.75)	169 (72.53)	155 (74.88)	71 (94.67)

**Table 3:** Multivariable logistic regression analysis for predictors of Incidental PCa after BPH surgery.

	Adjusted Odds Ratio	95% Confidence Interval	*p*-Value
**Age (years)**	1.02	1.021–1.028	<0.001
**Prostate MRI before surgery**	0.82	0.702–0.956	0.0114
**Prostate Biopsy before surgery**	0.74	0.654–0.840	<0.001
**MRI and Prostate Biopsy before surgery**	0.61	0.419–0.846	0.005
**TURP (reference)**	1	-	-
**HoLEP/ThuLEP**	0.68	0.598–0.773	<0.001
**Open Simple Prostatectomy**	1.16	1.020–1.326	0.0240
**Robot-Assisted Simple Prostatectomy**	0.79	0.639–0.969	0.0240

**Table 4:** Subgroup multivariable logistic regression analysis for predictors of Incidental PCa after BPH surgery in patients with preoperative biopsy or MRI.

	Adjusted Odds Ratio	95% Confidence Interval	*p*-Value
**Age (years)**	1.0094	0.995–1.023)	0.175
**TURP (reference)**	1	-	-
**HoLEP/ThuLEP**	0.87	(0.6037, 1.2657)	0.476
**Open Simple Prostatectomy**	1.25	(0.8091, 1.9312)	0.315
**Robot-Assisted Simple Prostatectomy**	0.70	(0.4047, 1.2363)	0.224

## Data Availability

The PearlDiver database is a private national database that requires private access to a password-protected server.
